# Eccentric hypertrophy impairs outcome after TAVR

**DOI:** 10.1007/s00392-024-02582-4

**Published:** 2024-12-09

**Authors:** R. Thalmann, V. Obermeier, Dominik S. Westphal, I. Diebold, T. Trenkwalder, C. Pellegrini, G. Buglio, H. Seoudy, P. Hoppmann, C. Bradaric, U. Schön, E. Holinski-Feder, N. Lettmann, H. Ruge, M. Erlebach, C. Fuetterer, K. L. Laugwitz, M. Krane, D. Frank, C. Kupatt

**Affiliations:** 1 Klinik Und Poliklinik Für Innere Medizin I, University Hospital Rechts Der Isar, School of Medicine and Health, Technical University of Munich, Ismaninger Str. 22, 81675 Munich, Germany; 2 DZHK (German Center for Cardiovascular Research), Partner Site Munich Heart Alliance, Munich, Germany; 3 Institute of Human Genetics, University Hospital Rechts Der Isar, School of Medicine and Health, Technical University of Munich, Munich, Germany; 4 MGZ Medizinisch Genetisches Zentrum Munich, Munich, Germany; 5 Technical University of Munich, Munich, Germany; 6 Cardiology, German Heart Centre Munich, School of Medicine and Health, Technical University Munich, Munich, Germany; 7 Klinik Für Innere Medizin III, University Hospital Schleswig-Holstein, Kiel, Germany; 8 DZHK (German Center for Cardiovascular Research), Partner Site Hamburg/Kiel/Lübeck, Kiel, Germany; 9 Department of Cardiovascular Surgery, Institute Insure, German Heart Center Munich, School of Medicine and Health, Technical University of Munich, Munich, Germany; 10 Institute of AI and Informatics in Medicine, University Hospital Rechts Der Isar, School of Medicine and Health, Technical University of Munich, Munich, Germany

**Keywords:** TAVR, Transcatheter aortic valve replacement, Hypertrophy, Outcome, Polygenic risk score, Cardiomyopathy

## Abstract

**Background:**

Aortic stenosis (AS) induces cardiac remodeling upon chronic left ventricular (LV) pressure overload. Here, we analyzed the clinical outcome of patients undergoing transcatheter aortic valve replacement (TAVR) for symptomatic AS with regard to varying LV hypertrophy patterns. Moreover, we investigated the genetic influence on development of different hypertrophy patterns, measured by polygenic risk scores (PRS).

**Methods:**

1703 patients with severe AS undergoing TAVR were categorized according to LV mass index and relative wall thickness in four subgroups: normal geometry (NG, *n* = 57), concentric remodeling (CR; *n* = 388), concentric hypertrophy (CH; *n* = 993) and eccentric hypertrophy (EH; *n* = 265). Data was analyzed retrospectively with regard to clinical outcome. In a substudy, 520 patients affected by CH (*n* = 237), EH (*n* = 139) or CR (*n* = 164) were analyzed using two PRS that have been previously associated with hypertrophic and dilated cardiomyopathy.

**Results:**

1 year after TAVR, for EH, in contrast to the remaining groups (NG, CR, CH), a significant difference in all-cause mortality was observable (mortality 17.4% EH, 14.0% NG, 12.4% CR, 14.0% CH, *p* = 0.001). This difference was observed up to 4 years (mortality 41.9% EH, 26.9% CH, 28.1% CR, 26.4% NG, *p* = 0.001). Of note, higher percentiles in a PRS for hypertrophic cardiomyopathy were associated with a reduced likelihood of EH in patients with AS (*p* = 0.046).

**Conclusions:**

The EH group had a statistically significant poorer 1-year and 5-year outcomes than the other groups. PRS might help predict myocardial reactions in patients with aortic stenosis in future.

**Supplementary Information:**

The online version contains supplementary material available at 10.1007/s00392-024-02582-4.

## Introduction

Aortic valve stenosis (AS) is the most prevalent heart valve disorder of aging populations in the western world [1]. AS causes chronic pressure overload of the left ventricle [2], increasing myocardial wall stress [3]. The increase in wall stress initiates mechanosensing and cellular adaption, i.e., formation of new sarcomeres, inducing cardiomyocyte hypertrophy in an attempt to counteract the increased wall stress and maintain cardiac output [4–6]. The primary adaptive response to the pressure overload caused by AS is concentric hypertrophy (CH) [7–10]. At advanced disease stages, cardiac CH is incapable to compensate the hemodynamic overload, and a systolic stress imbalance occurs associated with decrease in myocardial contractility and pump failure [4, 6]. Structural alterations of pathologic hypertrophy include interstitial fibrosis, capillary rarefaction, cardiomyocyte loss and declining contractile function [11]. At this stage, progression to eccentric hypertrophy (EH), sometimes referred to as “burn-out phase” [12], is suggested by previous reports [2, 6].

However, a majority of cases of dilated EH as end-stage AS do not seem to have undergone hypertrophic stages, previously. And vice versa: though a certain proportion of AS presented with concomitant atrial dilation, pulmonary hypertension and right ventricular failure, all indicating global and irreversible LV failure, most of them still present with hypertrophy [13]. The transition of a CH toward a dilatory state is rarely documented, confounders such as myocardial infarction excluded. This increases the likelihood of divergent reactions to identical stimuli: the heart either possesses the proper mechanosensing and sarcomere build-up signaling or it resumes to a less-responsive phenotype of cardiac dilation without previous hypertrophy.

Of inherited cardiomyopathies, such a divergence is well-known: particular intrinsic genetic defects may lead to hypertrophy of the cardiac muscle (hypertrophic cardiomyopathy (HCM), whereas other pathogenic variants induce a dilated cardiomyopathy (DCM). HCM and DCM are relatively common disorders, affecting 1:500 and 1:250 individuals, respectively [14, 15]. The identified pathogenic variants in HCM patients often affect genes that encode sarcomeric proteins such as myosin heavy chain (*MYH7*) or Titin (*TTN*) [14]. In the last years, however, disease-modifying variants have been identified that influence the occurrence risk of different cardiomyopathies in patients. The effect size of these common variants [i.e., single-nucleotide polymorphisms (SNP)] can be summarized in polygenic risk scores (PRS). PRS for the traits “hypertrophic myocardium” (HCM-PRS) [16] and “'Body surface area-indexed left ventricular end-systolic volume”, mirroring the DCM phenotype (DCM-PRS) [17] have been published before. As mentioned above, the influence of PRS on monogenic cardiomyopathies has already been shown, like the influence of the DCM-PRS on the DCM phenotype in carriers of truncation *TTN* variants [17]. Apart from that, PRS can be associated with risks in common diseases that are comparable to that of monogenic diseases [18].

In the current study, we have dichotomized AS patients according to CH and EH phenotypes and correlated them with clinical outcomes. Furthermore, based on the phenotypic dichotomy, we postulated that patients with EH, comparable to a DCM phenotype, would differ in their PRS percentiles from patients with a CH pattern in response to their acquired AS. To test this hypothesis, we examined this polygenic background by approximating the genetic impact on different AS hypertrophy patterns (CH, EH) using the previously published DCM-PRS and HCM-PRS.

## Methods

### Study design and population

The study was approved by local ethics committees and performed according to the Declaration of Helsinki. Written informed consent was obtained from all participating patients. This is a retrospective, multi-center, observational study. Patients with severe symptomatic aortic valve stenosis or combined vitium but leading stenosis who underwent transcatheter aortic valve replacement (TAVR) between 01/2014 and 08/2021 with sufficient baseline echocardiographic data were enrolled at the University Hospital rechts der Isar of the Technical University of Munich, the German Heart Center Munich and the University hospital Schleswig–Holstein, Kiel, Germany. Indication for TAVR was discussed individually based on age, frailty, co-morbidities, previous heart operations and present conditions by the local ‘Heart Team’ [19]. Preoperative clinical and laboratory examinations, transthoracic echocardiography (TTE), left heart catheterization, electrocardiogram, lung function test as well as CT angiography were performed in all patients before final decision. The procedure was performed under general anesthesia or analgosedation. Balloon-expandable or self-expanding valves were implanted. If the transfemoral approach was not possible, the transapical was used. Valve implantation was performed based on established protocols [20–23]. The choice between balloon-expandable and self-expandable valves was not made based on the predominant hypertrophy pattern. Postoperatively, prosthetic valve function was assessed via TTE. Clinical data at baseline as well as periprocedural and follow-up data were prospectively collected. Clinical data included age, gender, weight, height, body surface area (calculated using the DuBois formula), survival, date of procedure, date and cause of death, EuroSCORE I and II, comorbidities, cardiovascular risk factors, medical history, previous operations, cardiac, kidney and lung parameters as well as interventional parameters, echocardiographic measurements, initial symptoms and NYHA classification. Postinterventional survival data, echocardiographic data and NYHA classification were updated after 30 days, 3 month, 6 months, 1 year and thereafter every 6–12 months via visit or phone call.

**Fig. 1:**
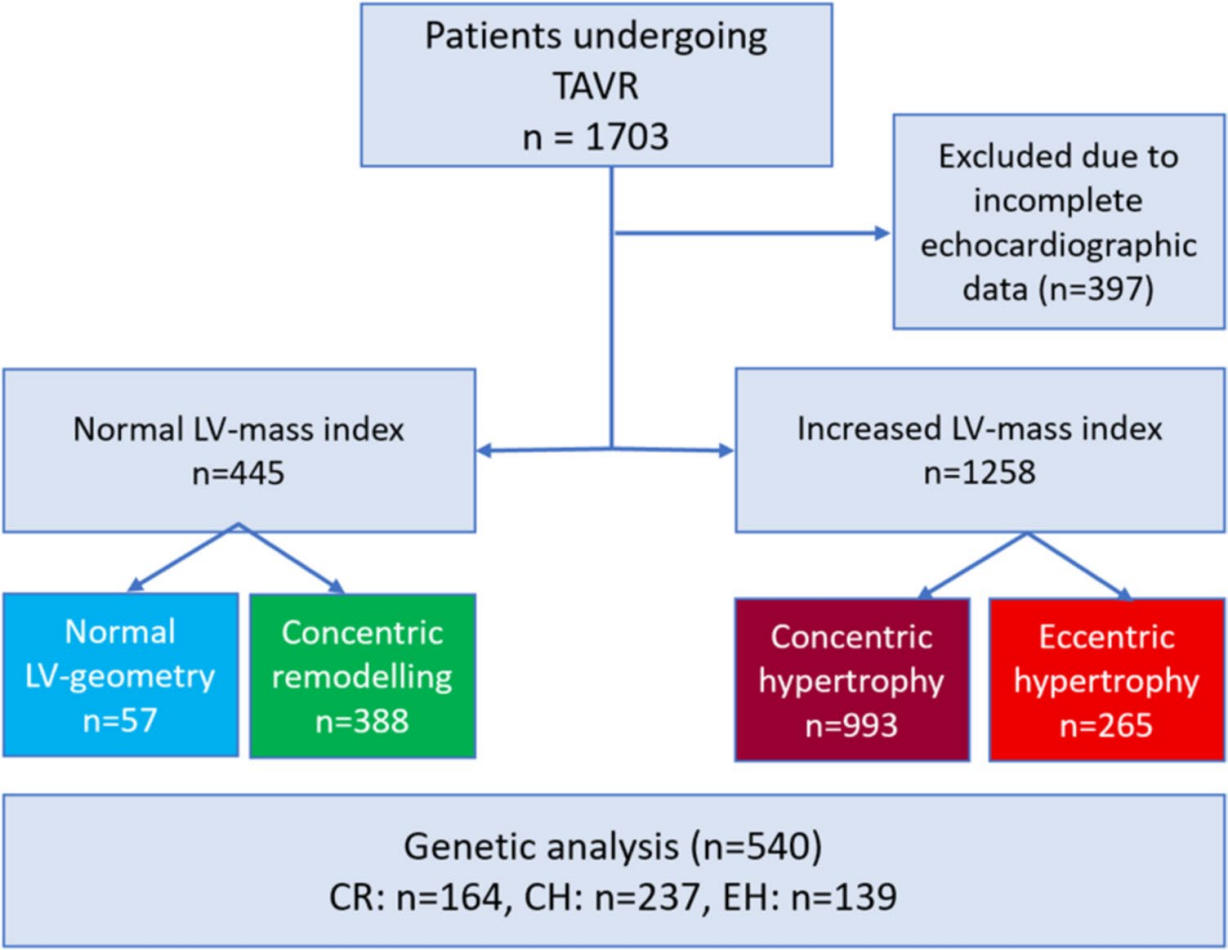
Flowchart depicting the study design and population. A total of 1703 patients that underwent transcatheter aortic valve replacement were included in this study in divided according to their hypertrophy pattern. A subgroup of 540 patients was included into the genetic analysis. *CH* concentric hypertrophy, *CR* concentric remodeling, *EH* eccentric remodeling, *LV* left ventricular

The collective registry included a total of 1703 patients who underwent TAVR. Based on the baseline echocardiographic data, relative wall thickness (RWT) and left ventricular mass index (LVMI) were calculated and the cohort was classified into four groups of hypertrophy pattern: normal geometry (NG), concentric remodeling (CR), concentric hypertrophy (CH) and eccentric hypertrophy (EH) (Fig. 1).

### Echocardiography

The echocardiogram was performed and evaluated by a team of single trained professionals conducting this on a regular base utilizing a commercially available echocardiography system equipped with a 2.5-MHz multifrequency phased array transducer. Echocardiographic measurements of the left ventricle were performed in consistence with the current recommendations for cardiac chamber quantification [24]. Left ventricular end-diastolic diameter (LVEDD), interventricular septum (IVS) and posterior wall thickness (PWT) were quantified in parasternal long-axis view using M mode or 2-dimensional echocardiography. Ejection fraction was determined quantitatively via the modified Simpson’s rules or qualitatively visual if the image quality was limited. AS as well as concomitant valve pathologies was graduated according to the current echocardiographic guidelines [25, 26]. LVMI was determined as LV mass divided by body surface area. The LV mass was calculated using the cube formula [24]: LV mass = 0.8 × 1.04 × [(IVS + LVEDD + PWT)^3^ − LVEDD^3^] + 0.6 g). RWT was calculated as (IVS + PWT)/LVID [24, 25] or (2 × PWT)/LVID [24]. According to LVMI and RWT, the cohort was categorized into the following four groups: normal geometry of the left ventricle was defined as normal RWT (≤ 0.42) and normal LVMI (LVMI ≤ 95 g/m^2^ for women and ≤ 115 g/m^2^ for men). In a left ventricle with CR, an elevated RWT (> 0.42) and a normal LVMI were found. Increased RWT and increased LVMI (LVMI > 95 g/m^2^ for women and > 115 g/m^2^ for men) were considered CH. Normal RWT of the left ventricular walls, including the septal region and the posterior wall, and raised LVMI was classified as EH.

### Endpoints

The primary outcome focused on overall survival after the TAVR procedure. Secondary endpoints involved adverse events as defined by the Valve Academic Research Consortium-2 (VARC-2 criteria) [27].

### Genetic analysis

As there is no PRS for the LV-hypertrophy in AS yet, two previously established PRS for the phenotypical correlated HCM [16] and DCM [17] were used in this study. Therefore, genome-wide single-nucleotide polymorphism (SNP) analysis was performed in DNA samples from a subgroup of 520 patients. EDTA blood samples were collected from patients from all three centers mentioned above. The samples underwent DNA extraction from blood lymphocytes following a standardized protocol. The Illumina SNP-Array (Illumina Inc., San Diego, USA) was used for the genotyping process which involved complementary binding of the patient's DNA to the nucleic acid sequences represented on the SNP-Array. Detection was achieved through fluorescence labeling captured using a laser camera of the Illumina iScan system (Illumina Inc., San Diego, USA). Allele frequencies for each SNP were calculated based on the genotyped data. PRS were computed using the calculated allele frequencies and SNP characteristics according to the algorithm developed by Allelica (Allelica Inc., New York, USA). This algorithm utilizes GWAS data calculating the already mentioned HCM-PRS [16] and DCM-PRS [17]. The results were distributed using percentiles and z scores. Reference values based on a validation study from the UK-biobank were derived by Allelica.

### Statistical analysis

Statistical analyses were performed using Microsoft Office Excel 2013 (Microsoft Corp., Redmond, WA, USA) and SPSS, version 29 (IBM, Chicago, USA). Post hoc comparison and multiple logistic regression were performed using R, version 4.3.1 (R Foundation for Statistical Computing, Vienna, Austria). Continuous variables are presented in the form mean ± standard deviation. For categorical variables, the percentages of the categories with absolute frequencies are given. For comparison between the groups, the Kruskal–Wallis test was performed for continuous variables and the Chi-Square or the Fisher’s exact test for categorical variables. In order to adjust for multiple testing, Bonferroni correction was applied to the obtained post hoc comparisons. Survival functions were estimated using the Kaplan–Meier method with log-rank testing. To identify predictors for all-cause mortality, all variables which showed a significant difference in survival in the univariate Cox model were included into the multivariable Cox regression.

To investigate the association of the DCM- and HCM-PRS percentile, the Pearson correlation with its 95% confidence interval [CI] was considered. To assess the genetic risk factors for EH and CH, a multiple logistic regression was performed. Additional cardiovascular risk factors, sex, arterial hypertension, renal insufficiency and dyslipidemia were taken into account using two multiple logistic regression models for modeling the occurrence of an EH and a CH adaption with reference to the hypertrophy category CR. A *p* value < 0.05 was considered as significant.

## Results

### Baseline characteristics and echocardiographic data

Between January 2015 and December 2022, 1703 patients underwent TAVR, received sufficient pre-TAVR echocardiography, and were included in the analysis. The mean age was 80 ± 6 years (range 35–97 years). Of these patients, 829 were male (49%). The patient cohort was divided into four groups according to their pre-TAVR hypertrophy pattern as described in the methods: NG was found in 57 patients (3.3%), CR in 388 patients (22.8%), CH in 993 patients (58.3%), and EH in 265 patients (15.6%).

Patients with EH had the highest logistic EuroSCORE I and II, and statistically significant differences were found between the groups CR versus CH and CR versus EH regarding EuroSCORE I and EuroSCORE II (*p* = 0.001). Compared to the other groups, more patients in the EH group were in NYHA-class III or IV and had a higher rate of left bundle branch blocks. The CH group had the highest proportion of women (61%). Statistically significant differences between the groups were found for logistic EuroSCORE I, II, higher NYHA classification, nicotine abuse, gender distribution, age, impaired renal function (GFR < 60 ml/min), previous myocardial infarction, and peripheral artery disease (Table 1). In a subgroup analysis of 527 patients, 8 (1.3%) additionally received a MitraClip in cases of severe mitral valve insufficiency prior to TAVI. There was no difference between the groups regarding the MitraClip rate. In a further subgroup analysis of 70 patients who underwent cardiac MRI, 5 (7.1%) showed pathological T1 relaxation times, suggesting possible cardiac amyloidosis. Regarding measured echocardiographic parameters, the highest mean aortic gradient was found in patients with CH, while the highest ejection fraction was measured in patients with CR. Patients with EH had the lowest preprocedural ejection fraction (43.2 ± 12.4%, *p* = 0.001), a low mean aortic gradient, and were associated with a high rate of higher-grade concomitant valve pathologies. Statistically significant differences between the groups were observed for ejection fraction and higher-grade aortic, mitral, and tricuspid valve insufficiency (Table 1, for all group comparisons via post hoc test, see the Supplementary Table).

**Table 1:** Baseline characteristics of the patients that were included into this study

Baseline characteristics	Total	Normal geometry	Concentric remodeling	Concentric hypertrophy	Eccentric hypertrophy	*p* value
Demographic, echocardiography and symptoms	*n* = 1703	*n* = 57	*n* = 388	*n* = 993	*n* = 265	
Age [yr]	80 ± 6	80 ± 6	80 ± 6	81 ± 6	79 ± 8	** < 0.001**
Sex [male]	829 (49)	39 (68)	255 (66)	384 (39)	151 (57)	** < 0.001**
Body mass index [kg/m^2^]	26.8 ± 5.0	27.0 ± 5.2	27.3 ± 4.9	26.6 ± 5.0	26.8 ± 7.9	0.104
Arterial hypertension	1510 (90)	50 (88)	344 (89)	879 (90)	237 (91)	0.816
Hypercholesterolemia	932 (56)	37 (65)	232 (60)	512 (53)	151 (58)	0.055
Diabetes mellitus	481 (29)	13 (23)	98 (26)	296 (30)	74 (29)	0.288
Impaired renal function	1182 (70)	42 (74)	219 (56)	746 (76)	175 (66)	** < 0.001**
Smoker	120 (11)	6 (15)	31 (12)	44 (8)	39 (16)	**0.002**
Coronary heart disease	1242 (74)	44 (77)	297 (77)	706 (72)	195 (75)	0.246
Previous myocardial infarction	219 (18)	14 (33)	29 (11)	118 (19)	58 (24)	** < 0.001**
Previous CABG	222 (13)	10 (18)	46 (12)	124 (13)	42 (16)	0.277
Atrial fibrillation	718 (43)	22 (39)	156 (41)	423 (43)	117 (45)	0.597
Left bundle branch block	138 (8)	4 (7)	15 (4)	85 (9)	34 (13)	** < 0.001**
Right bundle branch block	106 (6)	0 (0)	27 (7)	63 (6)	16 (6)	0.179
Peripheral artery disease	279 (17)	6 (11)	82 (22)	125 (13)	66 (26)	** < 0.001**
Chronic obstructive pulmonary disease	250 (15)	7 (13)	59 (16)	144 (15)	40 (16)	0.947
NYHA III / IV	1181 (72)	36 (64)	251 (66)	706 (74)	188 (75)	** < 0.001**
EuroSCORE I	17.5 ± 12.6	16.2 ± 12.3	15.4 ± 11.9	17.8 ± 12.3	20.0 ± 14.4	** < 0.001**
EuroSCORE II	5.9 ± 5.4	5.9 ± 5.3	5.4 ± 5.3	5.8 ± 5.0	7.1 ± 6.6	** < 0.001**
Left ventricular ejection fraction [%]	49.5 ± 9.8	46.5 ± 12.6	53.3 ± 6.7	49.9 ± 8.9	43.2 ± 12.4	** < 0.001**
Mean aortic gradient [mmHg]	36.8 ± 15.6	33.3 ± 12.5	36.2 ± 15.9	37.7 ± 16.1	34.8 ± 13.6	0.096
Mean septal wall thickness [mm]	13.6 ± 2.2	10.3 ± 2.6	12.5 ± 1.8	14.3 ± 2.0	12.6 ± 2.2	** < 0.001**
Aortic valve area [cm^2^]	0.77 ± 0.21	0.79 ± 0.21	0.77 ± 0.24	0.77 ± 0.19	0.79 ± 0.24	0.582
Aortic regurgitation ≥ °II	102 (7)	2 (4)	16 (5)	51 (6)	33 (16)	** < 0.001**
Mitral regurgitation ≥ °II	281 (17)	14 (25)	38 (10)	163 (17)	66 (25)	** < 0.001**
Tricuspidal regurgitation ≥ °II	207 (12)	10 (18)	38 (10)	127 (13)	32 (12)	** < 0.001**

Significances were marked as bold if they are beneath the significance level of *p* = 0.05

*CABG* coronary artery bypass graft, *CH* concentric hypertrophy, *CR* concentric remodeling, *EH* eccentric hypertrophy, *NG* normal geometry

### Procedural characteristics and postprocedural outcome

**Fig. 2:**
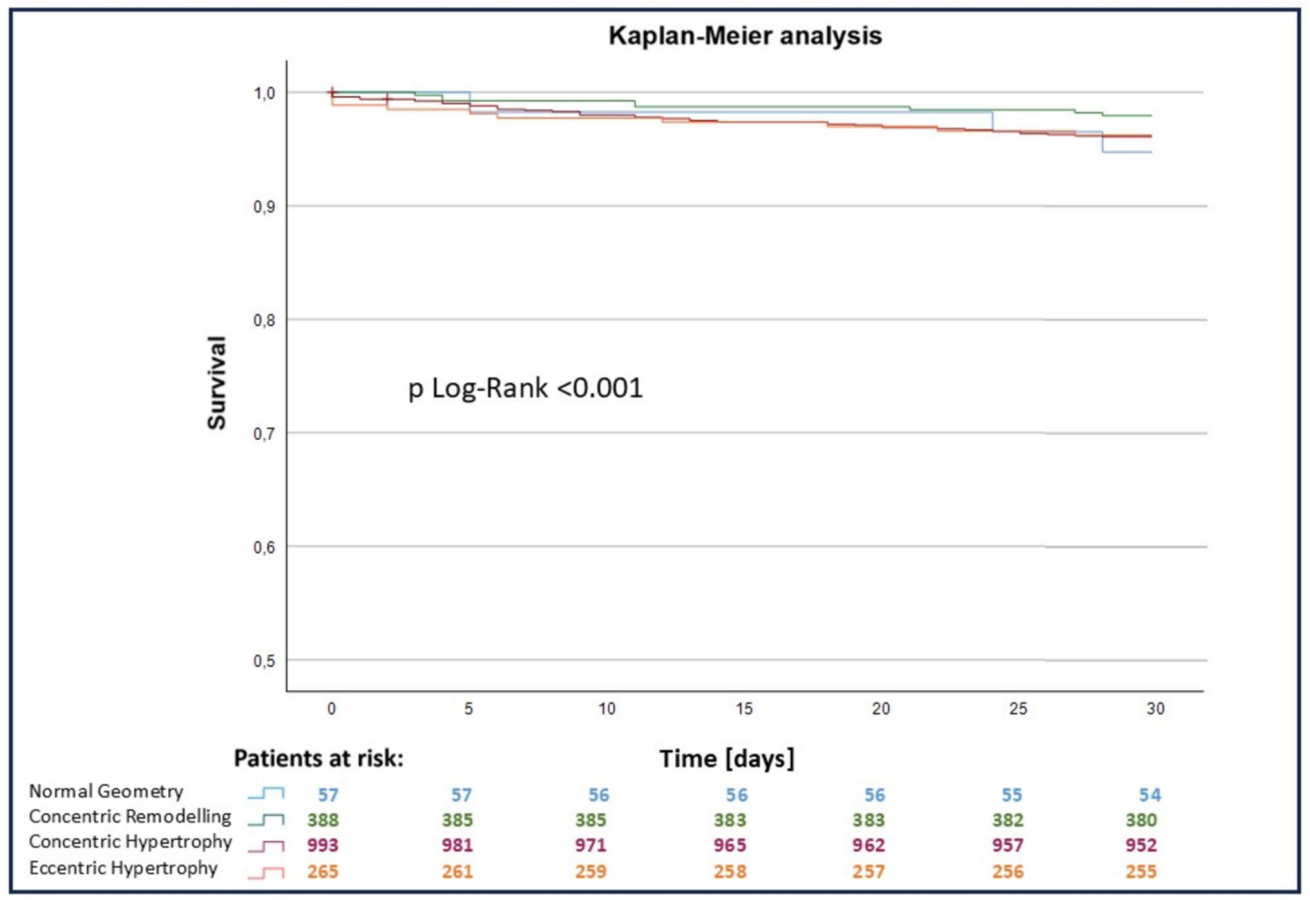
Kaplan–Meier estimates of the 30-day survival function after TAVR in the 4 groups determined by hypertrophy. Blue: patients with normal hypertrophy, green: patients with concentric remodeling, red: patients with concentric hypertrophy, orange: patients with eccentric hypertrophy

Most valves were implanted using the transfemoral approach. The most frequent implanted valves were the Edwards Sapien (57.4%) and the Medtronic CoreValve (41.1%). Self-expanding valves (SEV) were most frequent in the CH group (43%), while balloon expending valves (BEV) were mostly applied in patients with EH (74%) followed by CR (68%) and NG (67%). There were significantly more SEV in the CH group compared to EH (*p* < 0.001) and CR (*p* = 0.002). The average annulus size was 26.8 ± 3.0 mm. The overall 30-day all-cause mortality was 3.7%, with the highest proportion (*n* = 3, 5.3%) in the NG group (Fig. 2). 26 patients (1.6%) suffered from peri- or postoperative strokes: 1 patient in group NG (1.7%), 7 patients in group CR (1.8%), 14 patients in group CH (1.4%), and 4 patients in group EH (1.5%). 49 cases (3.0%) of major or life-threatening bleedings according to the VARC-2 criteria occurred. Within the first 72 h after TAVR, a myocardial infarction according to VARC-2 occurred in 6 patients (0.4%). Overall, 156 new pacemakers (9.5%) had to be implanted after the TAVR (NG 8.8%, CR 8.2%, CH 9.2% and EH 10.6%, respectively). The rate of new pacemakers after TAVR was 10.0% for SEV and 8.2% for BEV (*p* = 0.092).

### One-year and mid-term outcome

**Fig. 3:**
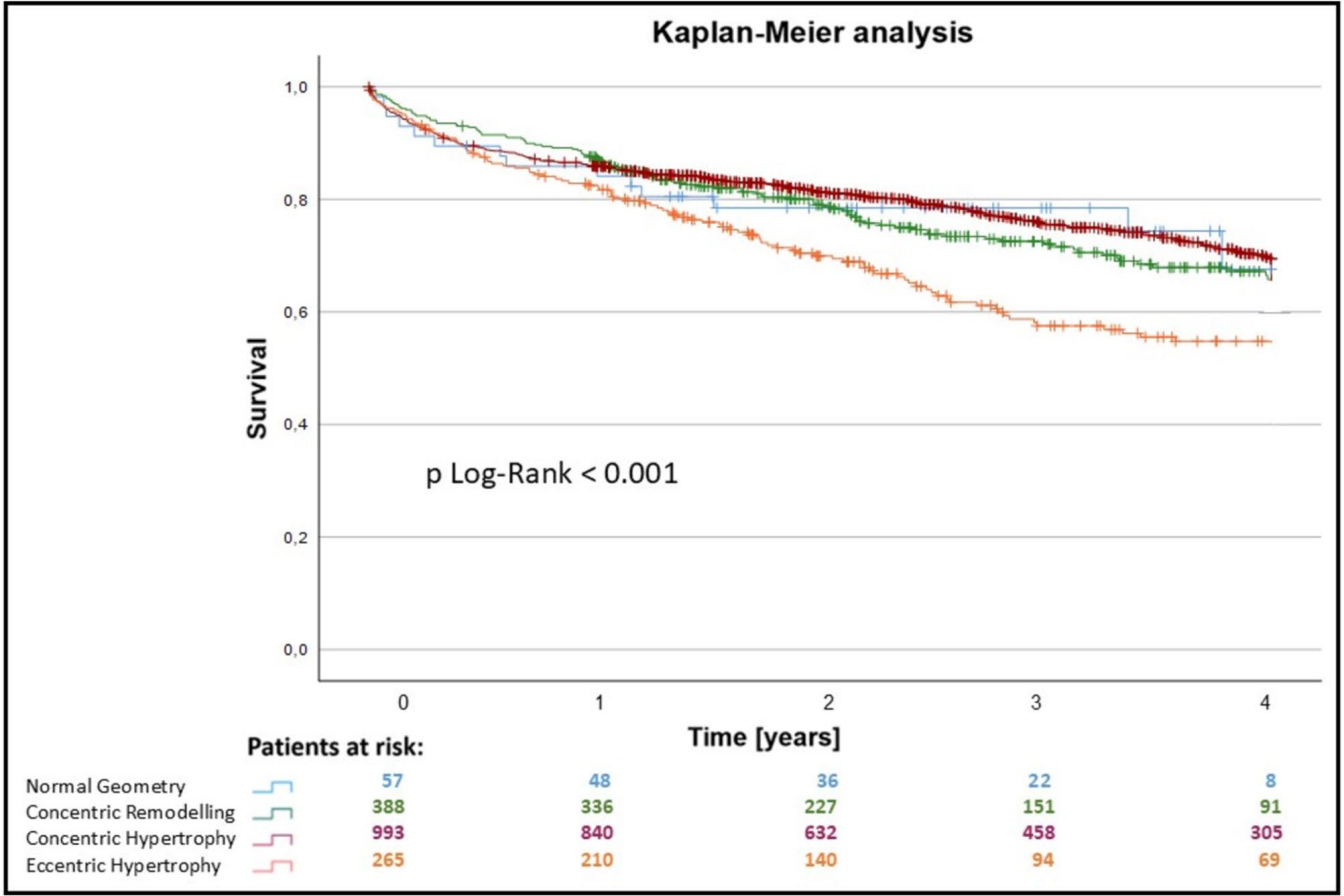
Mid-term survival after TAVR; Kaplan–Meier estimates of the survival function after TAVR in the 4 groups determined by hypertrophy. Blue: patients with normal hypertrophy, green: patients with concentric remodeling, red: patients with concentric hypertrophy, orange: patients with eccentric hypertrophy

Follow-up data for the patient cohort were collected up to seven years after TAVR. 397 patients were excluded due to missing data. A statistically significant difference between the groups regarding the postprocedural outcome was calculated for all-cause mortality after 30 days, 1 year and 4 years (*p* = 0.001 for 30 days, 1 year and 4 years) (Figs. 2 and 3). The overall mortality for SEV was significantly higher (38.1%) compared to BEV (20.7%, *p* < 0.001). Furthermore, the overall mortality was not significantly higher in patients with smaller annulus size (small annulus 35.9% versus 30.1%, *p* = 0.229).

At the 30 day timepoint, a statistical difference was found between the NG and CR groups, while no difference in survival between the CH and EH group was observable. The overall 1-year all-cause mortality rate was 14.2%. The EH group had the worst outcome in comparison the other groups (*p* < 0.001). Mid-term survival up to 4 years is illustrated in Fig. 3. Survival rates after 4 years were 73.6% (NG), 71.9% (CR), 73.1% (CH) and 58.1% (EH). The estimated survival functions are split according to the hypertrophy pattern groups.

### Multivariate analysis

A multivariate Cox regression was performed based on the significant results in the previously performed univariate regression. An association between all-cause mortality and age (odds ratio (OR): 1.030) as well as atrial fibrillation (OR: 1.576) was observed (*p* < 0.001). Moreover, preserved ejection fraction was associated with higher survival rates (OR: 0.973, *p* < 0.001, Table 2).

**Table 2:** Multivariate analysis of the significant influencing factors observed in the univariate regression

	B	Exp(B) (Odds Ratio)	*p* value
Age	**0.040**	**1.041**	** < 0.001**
Atrial fibrillation	**0.37**	**1.48**	** < 0.001**
Coronary artery disease	0.111	1.118	0.298
Hypercholesterolemia	− 0.170	0.844	0.065
Left ventricular ejection fraction	− **0.634**	**0.531**	** < 0.001**
NYHA ≥ III	0.007	1.000	0.917
Valve model	**0.502**	**1.653**	** < 0.001**

Significances were marked as bold if they are beneath the significance level of *p* = 0.05

*NYHA ≥ III* significant dyspnea at rest or during minimal activity according to the New-York-Hear-Association, *B* Beta-coefficient, *Exp (B)* odds ratio

### Genetic analysis

**Fig. 4:**
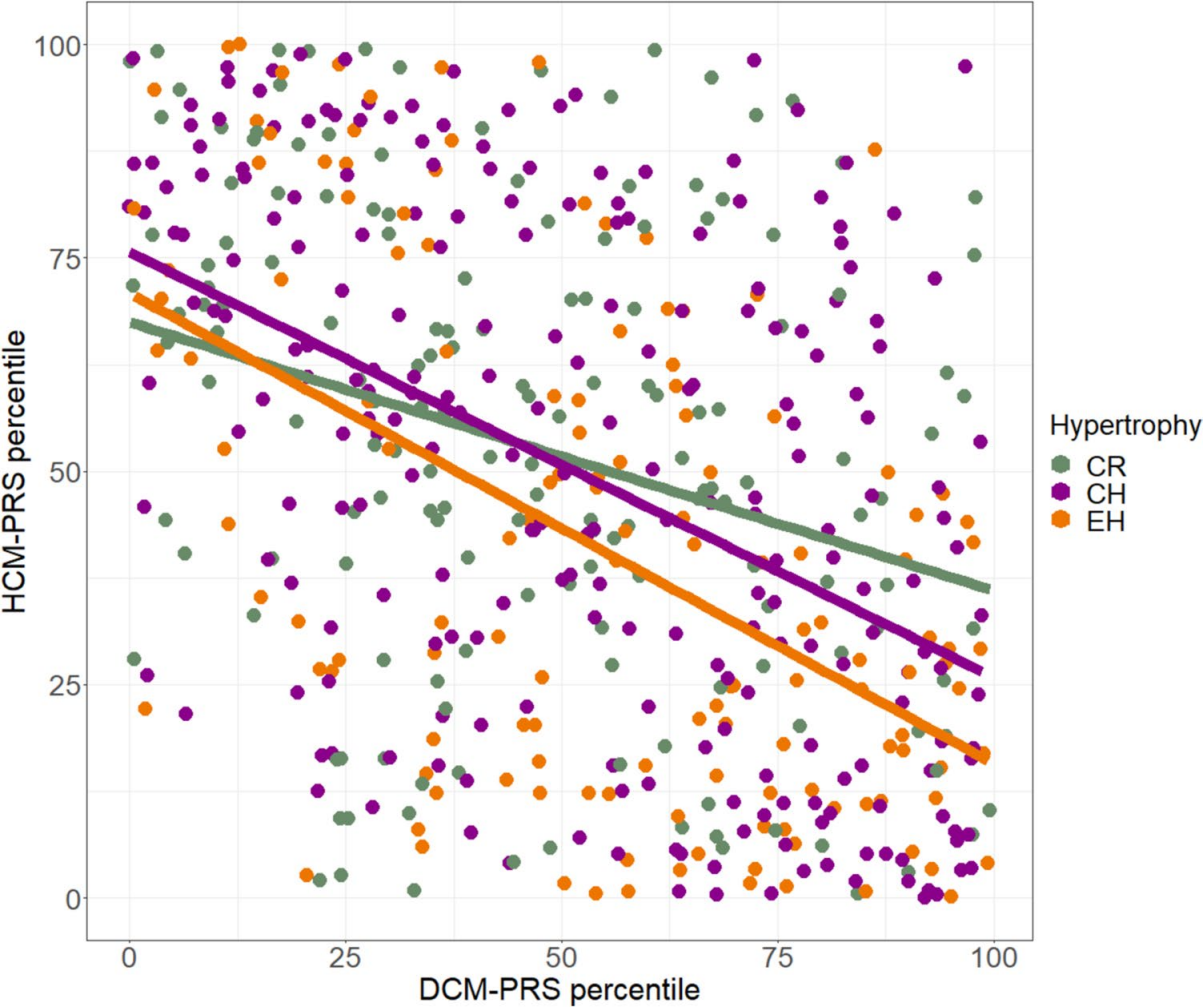
Scatterplot depicting the distribution of the measured PRS for DCM and HCM. There was an inverse correlation between the two measured polygenic risk scores in all three groups. Overall in patients with a high HCM-PRS value, low values for the DCM-PRS were observed. *CH* concentric hypertrophy (purple), *CR* concentric remodeling (green), *EH* eccentric remodeling (orange), *DCM-PRS* polygenic risk score for dilated cardiomyopathy, *HCM-PRS* polygenic risk score for hypertrophic cardiomyopathy

A subgroup of 540 AS cases with three distinct hypertrophic patterns (CR, CH, EH) were included in the genetic analysis (80.7 ± 6.2 years of age). 43.9% (*n* = 237) of patients displayed CH, while 25.7% (*n* = 139) demonstrated EH and 30.4% (*n* = 164) CR phenotypes. There was no statistically significant difference between the occurrence of CAD and arterial hypertension in these groups, which can be considered also causative for the EH or CH phenotype, respectively (Table 3). The percentile values of the polygenic risk scores (PRS) differed significantly in group comparisons. Particularly, the mean percentile values of hypertrophic cardiomyopathy polygenic risk scores (HCM-PRS) were considerably higher in the CH group when compared to the EH group (Mean percentiles ± standard deviation: CH = 49.5 ± 30.1, EH = 40.3 ± 29.3, *p* = 0.011). For all three hypertrophy categories, a negative Pearson correlation of the DCM-PRS and the HCM-PRS percentiles was observed, which was significantly different from zero. CR showed a weak inverse correlation of the DCM- and the HCM-PRS percentiles, which was—0.30 [− 0.44, − 0.16], *p* < 0.001, whereas EH and CH showed a moderate correlation of − 0.51 [− 0.63, − 0.38], *p* < 0.001 and − 0.47 [− 0.57, − 0.37], *p* < 0.001 (Fig. 4). This correlation has been described before [28] and could be reproduced in our cohort.

**Table 3:** Baseline characteristics of the genetic cohort

	CH (n = 237)	EH (n = 139)	*p* value
Age (mean ± SD)	81.8 ± 5.7	79.5 ± 7.5	**0.048**
Sex (male)—*n* (%)	94 (39.7)	87 (62.6)	** < 0.001**
CAD—*n* (%)	175 (73.8)	117 (84.1)	0.086
Art. hypertension—*n* (%)	221 (93.2)	131 (94.2)	0.624

Significances were marked as bold if they are beneath the significance level of *p* = 0.05

*CAD* coronary artery disease, *CH* concentric hypertrophy, *EH* eccentric hypertrophy

In a second step, cardiovascular risk factors, such as sex, arterial hypertension, renal insufficiency and dyslipidemia, were considered using two multiple logistic regression models for modeling the occurrence of a EH and CH with reference to CR. Concerning the prediction of hypertrophy EH versus CR, we can state that HCM-PRS had a significant influence (*p* = 0.046). There was an inverse correlation between the elevated HCM-PRS percentiles and the occurrence risk of EH (regression coefficient: − 0.011). However, the same difference in percentiles had no significant influence for CH compared to CR (*p* = 0.863). Moreover, no other PRS had a significant influence in none of the two models (Table 4).

**Table 4:** PRS values for the CH and EH subgroup

	PRS-HCM		PRS-DCM	
	CH	EH	CH	EH
Percentile value (mean ± SD)	52.5 ± 28.6	54.9 ± 27.3	49.5 ± 30.1	40.3 ± 29.3
	***p* = 0.038**		***p* = 0.011**	
Regression coefficient	− 0.0009	− 0.011	0.005	0.005
*p* value	0.863	**0.046**	0.333	0.381

Significances were marked as bold if they are beneath the significance level of *p* = 0.05

Inverse correlation between the elevated HCM-PRS percentiles and the occurrence risk of EH can be seen in the second column (*p* value = 0.046)

*CH* concentric hypertrophy. *CH* concentric hypertrophy, *EH* eccentric remodeling, *DCM-PRS* polygenic risk score for dilated cardiomyopathy, *HCM-PRS* polygenic risk score for hypertrophic cardiomyopathy

## Discussion

The present study revealed the impact of left ventricular hypertrophy patterns in patients with severe AS planned for TAVR on all-cause mortality after the procedure. Our retrospective study revealed that 1) patients presenting with a dilated ventricle (EH), who displayed a significantly lower EF, had the worst outcomes after 1 and 4 years and 2) that the occurrence of the EH phenotype was negatively correlated to the polygenic risk score for hypertrophic cardiomyopathy, i.e., at least partially genetically determined.

TAVR was initially developed for patients with AS without surgical treatment options. Therefore, the typical TAVR patient population is generally older, presenting with more comorbidities and a higher procedural risk compared to those receiving surgical aortic valve replacement [29, 30]. Our cohort reflects an intermediate- to high-risk TAVR profile according to Euro-Score-I and II. In our retrospective multi-center analysis, we included all-comers with AS receiving TAVR, irrespective of ejection fraction, comorbidities, or symptoms. Several studies have explored how AS influences LV remodeling. It has been observed that the primary adaptive response to the pressure overload caused by AS is concentric hypertrophy [7–10]. The EH patients, however, are potentially sicker and frailer compared to the other groups, possibly leading to the observed worse outcome in our study. This assumption is substantiated by statistically significant differences between EH and other groups regarding renal function, previous myocardial infarction, left bundle branch blocks and peripheral arterial disease as well as a higher EuroSCORE I and II. As described before in AS patients, a lower ejection fraction and a higher rate of mitral valve insufficiency in the EH group indicate a higher predisposition to heart failure [10].

The NG group exhibited a dismal 30-day and 1-year survival rate. Although the number of patients included in the NG group was relatively small, a high correlation between the rate of previous myocardial infarctions (33%) and a reduced EF and survival was observed. In these patients presenting with NG and impaired EF, a high mortality rate was found at 30d and 1 year (71.4% 1-year survival), whereas patients presenting with NG and a normal EF display an excellent survival (94.3% 1-year survival) Therefore, the NG group comprises two separate populations, one defined by, partially post-ischemic, functional deterioration and another with normal LV function, lacking a hypertrophic response. Other studies have found similar comorbidity (myocardial infarction NG = 44.4%) rates and procedural risks according to the Euro-SCORE I and II compared to our study [31].

Of note, for CH, an increased risk for cardiovascular morbidity and mortality has already been established in hypertensive patients [32]. In a study of 747 patients with AS and preserved ejection fraction, it was demonstrated that CH was independently associated with an increased risk of mortality selectively analyzing outcome in female patients [8]. Duncan et al. demonstrated that patients with a concentric hypertrophy had a worst outcome than those with a non-concentric LV pattern following SAVR. Additionally, they found that CR was associated with an increased risk of early death after surgery [33]. The negative prognostic impact of CR in patients with AS, regardless of medical or surgical management, was also demonstrated by another study [7] and observed in a TAVR cohort by Rymuza et al. [31]. The ejection fraction of the CR group in the study by Rymuza et al. was found to be higher than in the CH group [31]. Accordingly, in our study, CR also had a poorer outcome than CH at 4 years though the worst outcome was seen in the EH group. It remains puzzling why the CR cohort with a comparably well-preserved EF of 53.3 ± 6.7% is associated with a poorer mid-term survival compared to CH and NG.

Recently, PRS that were associated with the occurrence risk of AS were published in the literature [34, 35]. In order to predict the risk of adverse events in patients with AS, a PRS was established by Small et al. [36]. However, to the best of our knowledge, this study represents the first investigation of the correlation of PRS and left ventricular hypertrophy in calcific AS. We identified a correlation between the likelihood of occurrence of an EH pattern and a reduced PRS for hypertrophic cardiomyopathy when correcting for cardiovascular risk factors (*p* = 0.046). This finding might contribute to an improved understanding of the underlying pathomechanisms. However, there are limitations in the field of PRS as correlations do not necessarily imply causation, rather prediction, which may improve with evolution of the PRSs themselves [37]. With the improving predictive power of PRS, it is likely that small polygenic effects might be identified more precisely than currently possible. Tadros et al. demonstrated an inverse association between risk alleles of DCM and HCM [28]. In our study, only a negative association between the HCM-PRS and the occurrence of EH could be observed; conversely, no positive association was found between the DCM score and the incidence of EH. However, it is evident that the use of PRS generated from GWAS with patients with AS would be a more suitable approach.

## Limitations

High-quality echocardiography is an examination dependent on both patient constitution and the abilities of the examiner and can therefore vary. Strain echocardiography was not performed in this study but could be an interesting addition for future research to provide more detailed insights into myocardial function. There were very only limited numbers of patients included in the NG group, affecting the statistical power. The mean follow-up time was very limited with an average of only 2.82 years, which could reduce the accuracy of the long-term outcome data. No laboratory values, such as NT-proBNP, were included in the analysis as the negative prognostic impact had already been demonstrated in previous studies [38, 39]. Cardiac amyloidosis, a possible cause of left ventricular hypertrophy [40], could not be included as a prognostic factor due to the limited number of available cardiac MRI. Moreover, no specific tests like bone scan for detecting cardiac amyloidosis or light chain immunofixation for diagnosing amyloid light-chain (AL) amyloidosis were performed. Of note, PRS, which are calculated from GWAS in European individuals, have a lower predictive performance in non-European ancestry samples [41]. In this regard, our study was performed in a European population, which was the main source of variants in both genome-wide association studies used in this research [16, 17]. Another potential limitation is that the PRS were calculated for traits in patients suffering from cardiac hypertrophy caused by other pathologies than aortic stenosis. While it is most likely that alternative pathomechanisms exist in these patients, the measured traits are completely mimicked by the myocardial reaction in patients with aortic stenosis. The first trait, “hypertrophic cardiomyopathy”, measured by the PRS-HCM [16], also occurs in CH with elevated myocardial mass and a thickened wall [24]. The SNP heritability for HCM was about *h*^2^ = 34% in sarcomere-negative patients, indicating relatively high heritability [16]. Body surface area-indexed left ventricular end-systolic volume is the trait measured by the PRS-DCM. Its heritability is even higher with *h*^2^ = 43% [17]. This trait closely resembles the EH phenotype, characterized by an augmented left ventricular diameter. Therefore, we assume that it is appropriate to use these PRS in patients with AS although it is more likely an approximation.

## Conclusion

Compared to non-hypertrophic and concentric remodeling/hypertrophy patients, those presenting with aortic stenosis and eccentric hypertrophy exhibited a statistically significant inferior 1-year and 4-year outcomes. We found that polygenic risk scores may potentially assist in the prediction of eccentric hypertrophy as opposed to concentric hypertrophy in patients with aortic stenosis, rejecting the notion of a pathophysiologic continuum. Based on the findings of our study, specific PRS for LV-hypertrophy in AS and studies with larger sample sizes are needed in future to confirm the hypothesis that a reduced PRS-HCM might increase the risk for eccentric hypertrophy in AS.

## Supplementary Information

Below is the link to the electronic supplementary material.

Supplementary file1 (DOCX 25 kb)

## Data Availability

The data supporting the findings of this study are available from the corresponding author upon reasonable request.
