# Supplementary material for: Eccentric hypertrophy impairs outcome after TAVR
Source: Clin Res Cardiol. 2024 Dec 9;114(11):1516–26. doi: 10.1007/s00392-024-02582-4 (PMC12540619; doi:10.1007/s00392-024-02582-4)
Supplement: Supplementary file 1 — Supplementary file1 (DOCX 25 kb) [file 392_2024_2582_MOESM1_ESM.docx]

Supplement

Journal: Clinical Research in Cardiology

# Concentric versus excentric hypertrophy determine outcome after TAVR in patients with aortic stenosis

Thalmann R^1^*, Obermeier V^1,2^*, Westphal DS^1,2,3^*, Diebold I^4,5^, Trenkwalder T^2,6^, Pellegrini C^2,6^, Buglio G^1^, Seoudy H^7,8^, Hoppmann P^1^, Bradaric C^1^, Schoen U^4^, Holinski-Feder E^4^, Wolf D^4^, Lettmann N^1^,Ruge H^9^, Erlebach M^9^, Fuetterer C^10^, Schunkert H^2,6^, Laugwitz KL^1,2^, Krane M^9^, Frank D^7,8^, Kupatt C^1,2^

^1^ Klinik und Poliklinik für Innere Medizin I, University Hospital rechts der Isar, School of Medicine and Health, Technical University of Munich, Munich, Germany

^2^ DZHK (German Center for Cardiovascular Research), partner site Munich Heart Alliance, Munich, Germany^,^

^3^ Institute of Human Genetics, University Clinic rechts der Isar, School of Medicine and Health, Technical University of Munich, Munich, Germany

^4^ MGZ Medizinisch Genetisches Zentrum Munich, Munich, Germany

^5^ Technical University of Munich, Munich, Germany

^6^ Klinik für Kardiologie, German Heart Center Munich, Munich, Germany

^7^ Klinik für Innere Medizin III, University Hospital Schleswig-Holstein, Kiel, Germany

^8^ DZHK (German Center for Cardiovascular Research), partner site Hamburg/Kiel/Lübeck, Kiel, Germany

^9^ Klinik für Herz- und Gefäßchirurgie, German Heart Center Munich, Munich, Germany

^10^ Institute of AI and Informatics in Medicine, School of Medicine and Health, Klinikum rechts der Isar, Technical University of Munich, 81675 Munich, Germany

*Contributed equally

Corresponding author:

Dominik Westphal, Department of Internal Medicine I, Klinikum Rechts der Isar, School of Medicine and Health, Technical University of Munich, Ismaninger Str. 22, 81675 Munich, Germany. T +49 89 4140 2350. Email: [dominik.westphal@mri.tum.de](mailto:dominik.westphal@mri.tum.de)

# Supplementary Table 1: Post-hoc test for group comparison

| **Baseline characteristics** | ***p* Value** | **NG:CR** | **NG:CH** | **NG:EH** | **CR:CH** | **CR:EH** | **CH:EH** |
| --- | --- | --- | --- | --- | --- | --- | --- |
| **Post-hoc tests for group comparison** |  |  |  |  |  |  |  |
| **Age [yr]** | **< 0.001** | n.s. | 0.049 | n.s. | 0.009 | n.s. | < 0.001 |
| **Sex [male]** | **< 0.001** | n.s. | < 0.001 | n.s. | < 0.001 | n.s. | < 0.001 |
| **Body mass index [kg/m²]** | 0.104 | n.s. | n.s. | n.s. | n.s. | n.s. | n.s. |
| **Arterial hypertension** | 0.816 | n.s. | n.s. | n.s. | n.s. | n.s. | n.s. |
| **Hypercholesterolemia** | 0.055 | n.s. | n.s. | n.s. | n.s. | n.s. | n.s. |
| **Diabetes mellitus** | 0.288 | n.s. | n.s. | n.s. | n.s. | n.s. | n.s. |
| **Impaired renal function** | **< 0.001** | n.s. | n.s. | n.s. | < 0.001 | n.s. | 0.012 |
| **Smoker** | **0.002** | n.s. | n.s. | n.s. | n.s. | n.s. | 0.002 |
| **Coronary heart disease** | 0.246 | n.s. | n.s. | n.s. | n.s. | n.s. | n.s. |
| **Previous myocardial infarction** | **< 0.001** | 0.002 | n.s. | n.s. | 0.025 | 0.001 | n.s. |
| **Previous CABG** | 0.277 | n.s. | n.s. | n.s. | n.s. | n.s. | n.s. |
| **Atrial fibrillation** | 0.597 | n.s. | n.s. | n.s. | n.s. | n.s. | n.s. |
| **Left bundle branch block** | **< 0.001** | n.s. | n.s. | n.s. | 0.015 | < 0.001 | n.s. |
| **Right bundle branch block** | 0.179 | n.s. | n.s. | n.s. | n.s. | n.s. | n.s. |
| **Artery occlusive disease** | **< 0.001** | n.s. | n.s. | n.s. | < 0.001 | n.s. | < 0.001 |
| **Chronic obstructive pulmonary disease** | 0.947 | n.s. | n.s. | n.s. | n.s. | n.s. | n.s. |
| **NYHA III / IV** | **< 0.001** | n.s. | n.s. | n.s. | < 0.001 | n.s. | < 0.001 |
| **Logistic EuroSCORE I** | **< 0.001** | n.s. | n.s. | n.s. | < 0.001 | < 0.001 | n.s. |
| **EuroSCORE II** | **< 0.001** | n.s. | n.s. | n.s. | n.s. | < 0.001 | n.s. |
|  |  |  |  |  |  |  |  |
| **Left ventricular ejection fraction [%]** | **< 0.001** | 0.003 | n.s. | n.s. | < 0.001 | < 0.001 | < 0.001 |
| **Mean aortic gradient [mmHg]** | 0.096 | n.s. | n.s. | n.s. | n.s. | n.s. | n.s. |
| **Mean septal wall thickness [mm]** | **<0.001** | <0.001 | <0.001 | <0.001 | <0.001 | 0.334 | <0.001 |
| **Aortic valve area [cm²]** | 0.582 | n.s. | n.s. | n.s. | n.s. | n.s. | n.s. |
| **Aortic regurgitation ≥ °II** | **< 0.001** | n.s. | n.s. | n.s. | 0.001 | 0.005 | n.s. |
| **Mitral regurgitation ≥ °II** | **< 0.001** | < 0.001 | n.s. | n.s. | < 0.001 | < 0.001 | n.s. |
| **Tricuspidal regurgitation ≥ °II** | **< 0.001** | < 0.001 | n.s. | n.s. | < 0.001 | n.s. | n.s. |

Supplementary Table: Post-hoc test for group comparison (n.s. = not significant)

**Supplementary Table 2: Univarate Regression**

|  | **B** | **Exp(B)** | ***p* value** |
| --- | --- | --- | --- |
| **Age [yr]** | **0.009** | **0,126** | **<0.001** |
| **Sex [male]** | -0.222 | 0.801 | 0.222 |
| **Body mass index [kg/m²]** | -0.002 | -0.220 | 0.434 |
| **Arterial hypertension** | -0.289 | 0.749 | 0.300 |
| **Hypercholesterolemia** | **-0.434** | **0.648** | **0.020** |
| **Diabetes mellitus** | 0.106 | 1.111 | 0.592 |
| **Impaired renal function** | 0.002 | 1.002 | 0.998 |
| **Smoker** | 0.180 | 1.197 | 0.551 |
| **Coronary heart disease** | **0.507** | **1.661** | **0.011** |
| **Previous myocardial infarction** | -0.003 | 0.997 | 0.988 |
| **Previous CABG** | -0.331 | 0.718 | 0.227 |
| **Atrial fibrillation** | **0.719** | **2.052** | **<0.001** |
| **Left bundle branch block** | 0.152 | 1.164 | 0.065 |
| **Right bundle branch block** | 0.072 | 1.075 | 0.904 |
| **Artery occlusive disease** | 0.096 | 1.101 | 0.677 |
| **Chronic obstructive pulmonary disease** | 0.050 | 1.051 | 0.837 |
| **NYHA III / IV** | **0.605** | **1.832** | **0.004** |
| **Logistic EuroSCORE I** | 0.0001 | 0.058 | 0.060 |
| **EuroSCORE II** | 2,79x10^-5^ | 0.008 | 0.785 |
| **Valve model** | **0.489** | **1.631** | **<0.001** |
|  |  |  |  |
| **Left ventricular ejection fraction [%]** | **-0.008** | **-0.182** | **<0.001** |
| **Mean aortic gradient [mmHg]** | 0.0001 | 0.049 | 0.109 |
| **Aortic valve area [cm²]** | 9.04x10^-5^ | 0.022 | 0.475 |
| **Aortic regurgitation ≥ °II** | 0.022 | 1.023 | 0.946 |
| **Mitral regurgitation ≥ °II** | -0,143 | 0.867 | 0.570 |
| **Tricuspidal regurgitation ≥ °II** | -0,177 | 0.838 | 0.546 |

*Supplementary Table 2:* *Univariate analysis of the influencing factors. (NYHA ≥ III: significant dyspnea at rest or during minimal activity according to the New-York-Hear-Association, CABG: coronary artery bypass graft, B: Beta-coefficient, Exp (B): Odds-Ratio)*
